# Outer Membrane Porin F in E. coli Is Critical for Effective Predation by *Bdellovibrio*

**DOI:** 10.1128/spectrum.03094-22

**Published:** 2022-11-29

**Authors:** Wonsik Mun, Sumudu Upatissa, Sungbin Lim, Mohammed Dwidar, Robert J. Mitchell

**Affiliations:** a School of Biological Sciences, Ulsan National Institute of Science and Technology (UNIST), Ulsan, South Korea; b Cleveland Clinic Lerner College of Medicine, Case Western Reserve University, Cleveland, Ohio, USA; c Department of Cardiovascular & Metabolic Sciences, Lerner Research Institute, Cleveland Clinic, Cleveland, Ohio, USA; d Center for Microbiome and Human Health, Lerner Research Institute, Cleveland Clinic, Cleveland, Ohio, USA; Emory University

**Keywords:** predatory bacteria, bdellovibrios, predation, outer membrane proteins

## Abstract

*Bdellovibrio* and like organisms (BALOs) are a unique bacterial group that live by predating on other bacteria, consuming them from within to grow and replicate before the progeny come out to complete the life cycle. The mechanisms by which these predators recognize their prey and differentiate them from nonprey bacteria, however, are still not clear. Through genetic knockout and complementation studies in different Escherichia coli strains, we found that Bdellovibrio bacteriovorus strain 109J recognizes outer membrane porin F (OmpF) on the E. coli surface and that the activity of the E. coli EnvZ-OmpR regulatory system significantly impacts predation kinetics. OmpF is not the only signal by which BALOs recognize their prey, however, as B. bacteriovorus could eventually predate on the E. coli *ΔompF* mutant after prolonged incubation. Furthermore, recognizing OmpF as a prey surface structure was dependent on the prey strain, as knocking out OmpF protein homologues in other prey species, including Escherichia fergusonii, Klebsiella pneumoniae, and Salmonella enterica, did not always reduce the predation rate. Consequently, although OmpF was found to be an important surface component used by Bdellovibrio to efficiently recognize and attack E. coli, future work is needed to determine what other prey surface structures are recognized by these predators.

**IMPORTANCE** Bdellovibrio bacteriovorus and like organisms (BALOs) are Gram-negative predatory bacteria that attack other Gram-negative bacteria by penetrating their periplasm and consuming them from within to obtain the nutrients necessary for the predator’s growth and replication. How these predators recognize their prey, however, has remained a mystery. Here, we show that the outer membrane porin F (OmpF) in E. coli is recognized by B. bacteriovorus strain 109J and that the loss of this protein leads to severely delayed predation. However, predation of several other prey species was not dependent on the recognition of this protein or its homologues, indicating that there are other structures recognized by the predators on the prey surface that are yet to be discovered.

## OBSERVATION

In nature, microbes employ a range of approaches to survive. One group of bacteria that have evolved an incredibly unique, yet successful, lifestyle is the *Bdellovibrio* and like organisms (BALOs), Gram-negative bacterial strains that attack and consume other Gram-negative strains (1, 2). The best-characterized BALOs employ an intraperiplasmic growth phase, where they enter the periplasm of the prey and secrete numerous hydrolytic enzymes (3, 4). These enzymes hydrolyze the prey’s proteins and nucleic acids (5, 6) to generate the monomers necessary for the BALOs’ growth and replication. Although research during the last 2 decades has done much to unravel the life cycle of BALOs, many mysteries still surround these predators, including how these predators recognize their prey.

When they were originally isolated in 1962 (7), BALOs were thought to be slow-growing bacteriophages, as their activities closely mimic those of bacterial viruses. Bacteriophages that infect Gram-negative microbes have evolved a variety of means to infect their bacterial hosts, including the targeting of different receptor proteins within the host’s outer membrane (8, 9). Given the comparable activities of predatory bacteria and phages, we were curious as to whether BALOs use similar host protein cell receptors as phages do to recognize their prey. This was explored using several isogenic mutants of E. coli strain BW25113, which lacks known phage receptor proteins (Table S1 in the supplemental material). Our panel included mutants with mutations in the FepA, FadL, FhuA, and OmpF outer membrane proteins, which serve as receptors for a wide range of phages (10–13), as well as TonB, which is required by some phages for their transport across the outer membrane (10).

**FIG 1:**
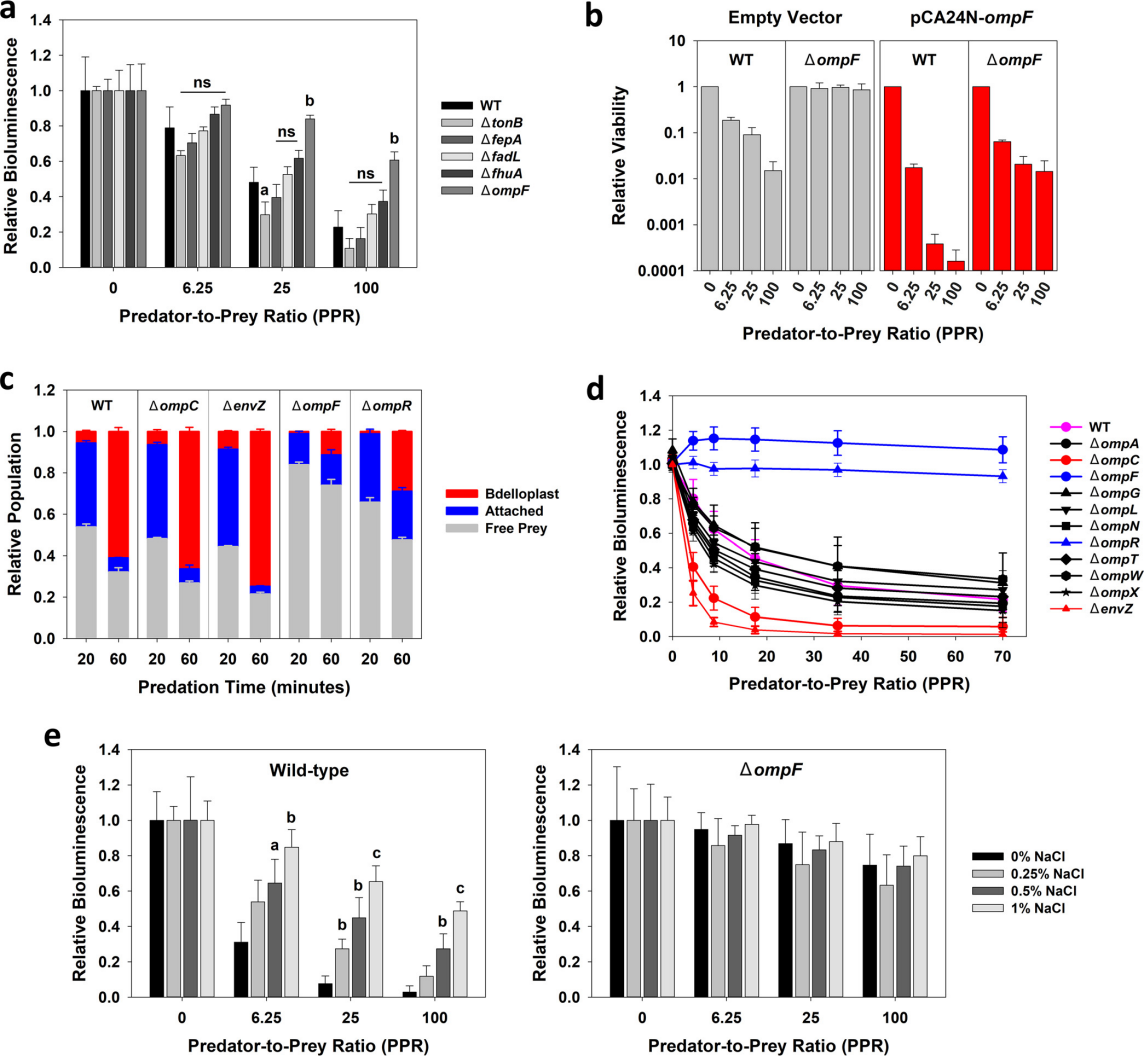
Loss of OmpF in E. coli significantly impacts predation rates. (a) Predation of various phage receptor mutants in E. coli strain BW25113. The relative bioluminescence results (determined at 1 h) show that predation was severely delayed when *ompF* was knocked out. Statistical significance was determined against the wild-type (WT) E. coli BW25113 response. ns, not significant; a, *P < *0.05; b, *P < *0.01. Error bars show standard deviations (SD) (*n *=* *3). (b) Expression of a functional *ompF* gene increases predation rates. Complementation of the Δ*ompF* knockout led to similar predation rates as in the wild-type E. coli BW25113, while overexpression of the *ompF* gene in the wild-type E. coli BW25113 background led to significantly better predation rates. The viability was measured after 1 h of predation. Error bars show SD (*n *=* *3). (c) Loss of *ompF* hinders attachment of the predator to the prey and delays bdelloplast development. The numbers of each population (i.e., free prey, prey having a predator attached, or bdelloplast) were determined for each genetic background at 20 and 60 min after initiating predation. The results show that loss of *ompC* or *envZ* slightly increased attachment and bdelloplast formation, while loss of *ompF* or *ompR* had the opposite impact at both time points. An average of >100 bacterial cells was used to analyze each independent sample. Error bars show SD (*n *=* *3). (d) Impact of other outer membrane proteins on predation rates. Eleven different isogenic mutants (Table S1) were evaluated based on their relative bioluminescence values. The loss of none of these genes had a significant impact on predation rates other than those related to the EnvZ-OmpR two-component regulatory system, i.e., *envZ*, *ompR*, *ompC*, and *ompF*. Error bars show SD (*n *=* *3). (e) Growth of E. coli at higher osmolalities reduces its predation rate. The osmolality of the growth medium was adjusted based on the NaCl content (0 to 1% [wt/vol]). The results show that growth of wild-type E. coli BW25113/pGen-luxCDABE at higher osmolalities decreased its predation (left), while the absence of a functional *ompF* gene prevented this; i.e., each culture was predated equally regardless of the growth medium’s osmolality (right). Statistical significance was determined against the 0% NaCl culture responses. a, *P < *0.05; b, *P < *0.01; c, *P < *0.001. Error bars show SD. (*n *=* *3).

As shown by the results in Fig. 1a, using prey bioluminescence as a proxy for predation (14, 15), all the isogenic mutants had susceptibilities that were like those of wild-type E. coli BW25113, except E. coli strain JW0912 (Δ*ompF*), which was significantly more resistant. The results in Fig. S1 and S2 show that this strain was eventually predated, however, suggesting that the invasion of the predator was delayed but not totally inhibited. Comparable results were also reported by Maffei et al. (16) in their study with bacteriophages within *Myoviridae* subfamily *Tevenvirinae*, which had significantly reduced but perceptible activities against isogenic *ompF* mutants of E. coli. Moreover, complementing Δ*ompF* in E. coli JW0912 restored this strain’s susceptibility to predation, while overexpression of *ompF* in wild-type E. coli BW25113 increased its predation kinetics (Fig. 1b and Fig. S3), affirming the importance of this outer membrane protein (OMP) to predation.

Based on the data in Fig. 1c and Fig. S4, OmpF is likely a receptor to recognize this prey, as nearly half (46.1%) of E. coli BW25113 cells had predators attached to them or were bdelloplasts after only 20 min. In contrast, for the isogenic mutant E. coli JW0912 (Δ*ompF*), only 16.6% of the population had predators attached or within them, showing that attachment was significantly lower for this mutant E. coli strain. This difference was exacerbated further at 1 h, when 61.0% of E. coli BW25113 cells were bdelloplasts, as opposed to only 11.3% of E. coli JW0912 (Δ*ompF*) cells (Fig. 1c), and the difference in rates of predation was not due to any obvious differences in the prey cell densities (Fig. S5), proving that a loss of OmpF delays predation and that this protein is likely being used by the predator to recognize E. coli.

Stemming directly from this discovery, the importance of other outer membrane porins (OMPs) in E. coli was also evaluated. Among the eight additional strains tested, only one led to a meaningful change in the predation kinetics: an *ompC* mutant (Fig. 1d) that made the prey more susceptible to predation, as when *ompF* was overexpressed (Fig. 1b). When *ompC* was complemented, the strain was less susceptible (Fig. S3), demonstrating that OmpC expression had the opposite impact as OmpF. This inverse relationship between Δ*ompF* and Δ*ompC* led us to test the involvement of the EnvZ-OmpR two-component regulatory system. Within E. coli, EnvZ acts as an inner membrane sensory protein that responds to changes in the medium’s osmolality and transmits this information to the transcriptional regulator OmpR, forcing it to take one of two alternative structures that positively regulate the expression of either OmpC or OmpF (17). Previous studies found that OmpF expression is dependent on the presence of OmpR (17–19), while EnvZ is required for the maximum production of OmpC (17, 20). Consistent with those reports, loss of the *ompR* gene led to predation resistance (similar to the effect of the Δ*ompF* mutation), while the Δ*envZ* mutant showed enhanced susceptibility to predation, like what was observed with the Δ*ompC* mutant (Fig. 1c and d). However, as shown by the results in Fig. S6, OmpF is crucial for predation, as its expression in a Δ*ompC* Δ*ompF* double mutant restored susceptibility.

As noted above, the EnvZ-OmpR two-component regulatory system recognizes increases in the medium’s osmolality, leading to the repression of *ompF* transcription (17, 21). Consequently, we explored whether growth of E. coli at different osmolalities would impact its susceptibility to predation. As shown by the results in Fig. 1e, this was the case, with clear dose-dependent responses according to the medium’s osmolality; i.e., growing E. coli overnight in LB medium with high concentrations of NaCl reduced predation rates against this prey population. In contrast, no greater resistance was observed when E. coli JW0912 (Δ*ompF*) was grown at the higher osmolalities (Fig. 1e). Moreover, tests with the Δ*ompR* and Δ*envZ* strains found that the osmolality had no impact on the susceptibility of E. coli strain JW3368 (Δ*ompR*), which remained resistant, but had a slight impact on the susceptibility of E. coli strain JW3367 (Δ*envZ*) (Fig. S7). It is known that OmpR-mediated osmoregulation of *ompC* and *ompF* can occur independent of EnvZ (22), as this response regulatory protein can respond in a noncanonical manner when *envZ* is deleted (23). As such, the differential responses observed with E. coli JW3367 (Δ*envZ*) might be related to these other regulatory principles. Taken together, these findings once more identify OmpF as a crucial surface protein for prey recognition but also point toward the E. coli EnvZ-OmpR two-component regulatory system as being a critical factor controlling susceptibility.

**FIG 2:**
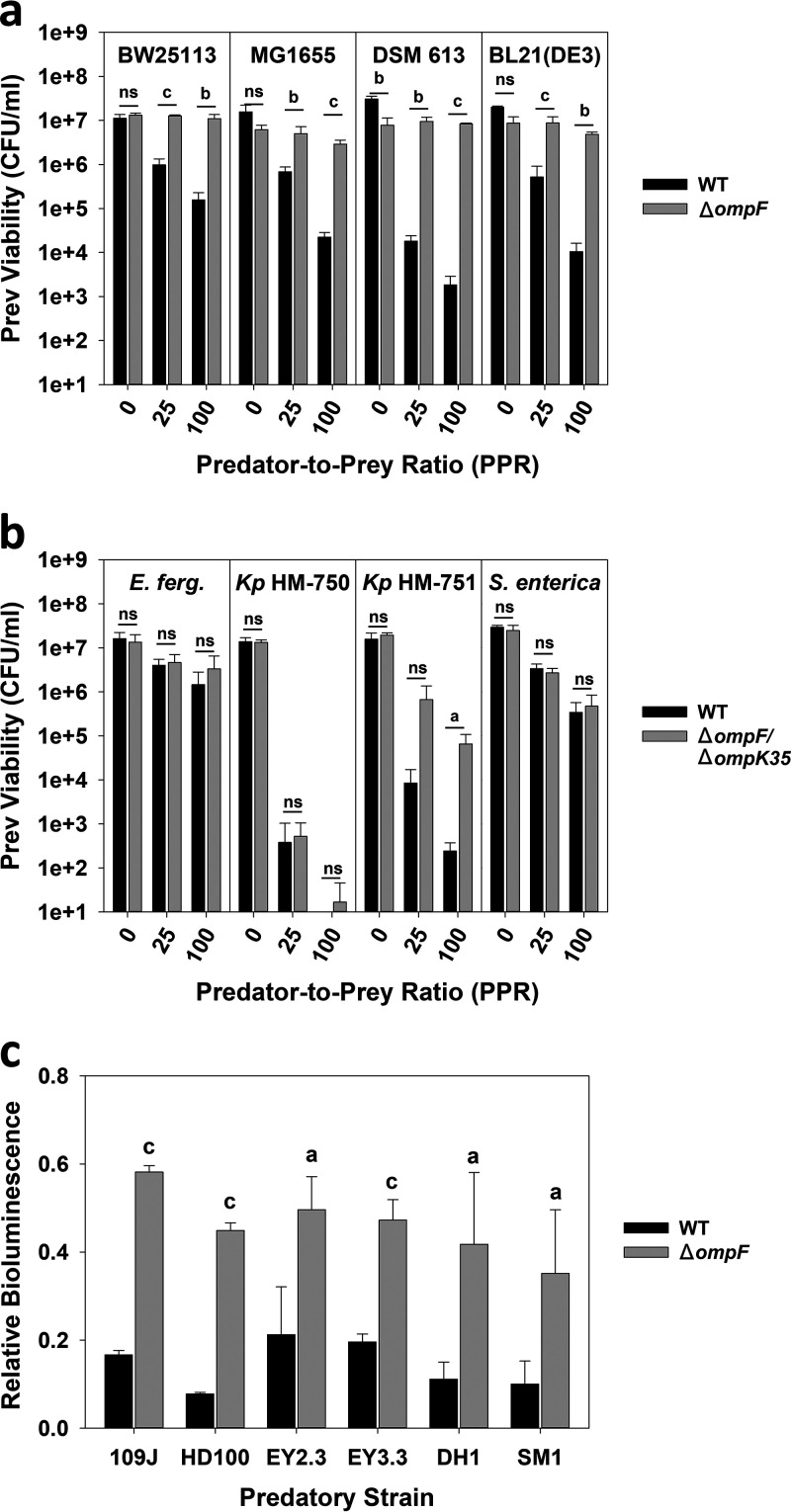
OmpF is important in the recognition of E. coli but not for other prey species. (a) Loss of OmpF in various E. coli strains inhibited predation by B. bacteriovorus strain 109J. The prey strains evaluated included both K-12 and B strains of E. coli. Statistical significance was determined against the wild-type response. ns, not significant; b, *P < *0.01; c, *P < *0.001. Error bars show SD (*n *=* *3). (b) Loss of OmpF or its homologues had varied impacts with other prey species. Within both E. fergusonii and S. enterica, the loss of OmpF had no obvious impact, while for K. pneumoniae, it depended on the strain. While the predation rates were identical for K. pneumoniae strain WGLW1 (HM-750) and its isogenic *ompK35* knockout mutant, loss of the *ompK35* gene in K. pneumoniae strain WGLW2 (HM-751) delayed predation of this pathogen by B. bacteriovorus 109J. Statistical significance was determined against the wild-type response. ns, not significant; a, *P < *0.05. Error bars show SD (*n *=* *3). (c) Loss of OmpF in E. coli BW25113 impacts its predation by various *Bdellovibrio* strains. Six different Bdellovibrio strains were evaluated, including two new isolates obtained from a wastewater treatment plant (strains EY2.3 and EY 3.3) and two from forest soil (strains DH1 and SM1). In each case, the absence of OmpF significantly delayed predation. Statistical significance was determined against the wild-type response. a, *P < *0.05; c, *P < *0.001. Error bars show SD (*n *=* *3).

The importance of OmpF for B. bacteriovorus strain 109J predation in other prey strains and species was then explored. As shown by the results in Fig. 2a, loss of the *ompF* gene led to resilient phenotypes (2- to 4-log better survival) in three additional E. coli strains, including both K-12 (MG1655) and B strains [BL21(DE3) and DSM 613], proving that this gene and its protein regulate prey recognition within E. coli. In contrast, when other bacterial species were tested, the results were not as clearly defined. As shown by the results in Fig. 2b, loss of the *ompF* homologue (Fig. S8) in Escherichia fergusonii strain ATCC 35469 and Salmonella enterica strain LT2 did not significantly affect their predation rates by B. bacteriovorus 109J. This was also the case for Klebsiella pneumoniae strain WGLW1 (HM-750) when its *ompK35* gene (homologue of *ompF*) was deleted. Tests with another strain of K. pneumoniae (strain WGLW2 [HM-751]), however, found that this prey was slightly resilient when its *ompK35* gene was deleted (Fig. 2b), even though the amino acid sequences for both OmpK35 proteins were identical (Fig. S8). Moreover, introducing a replicating plasmid expressing E. coli K12 ompF (pCA24N-*ompF*) into E. fergusonii ATCC 35469 or its isogenic ompF knockout mutant slightly increased its susceptibility to predation (Fig. S9).

As such, OmpF acts as a receptor for B. bacteriovorus 109J in recognizing E. coli prey, and to a lesser extent, K. pneumoniae, but is clearly not used universally for all susceptible bacterial species, implying that other components/proteins must also serve as receptors for this predator. This helps explain why E. coli JW0912 (Δ*ompF*) is eventually predated (Fig. S2), i.e., a single receptor is not used to recognize any given prey. This is known to be true for bacteriophages, as both lipopolysaccharides (LPS) and affinity transporters can also serve as receptors (24). Relatedly, Schelling and Conti reported that the LPS core was used as a receptor in S. enterica LT2 for B. bacteriovorus strain 109D (25), potentially explaining why OmpF was not required for this prey. As similar work has not been explored in E. coli, however, the importance of the LPS as a receptor in this prey should be investigated.

Since B. bacteriovorus 109J clearly uses OmpF to recognize E. coli, we were curious as to whether other predatory strains use the same receptor. To explore this, the activities of several additional BALO strains, including B. bacteriovorus strain HD100, two Bdellovibrio isolates obtained from a local wastewater treatment plant (strains EY2.3 and EY3.3), and two from forest soil (strains DH1 and SM1) (Table S3), were evaluated. As shown by the results in Fig. 2c, predation of E. coli JW0912 (Δ*ompF*) was significantly delayed compared to the predation of wild-type E. coli BW25113 for all six Bdellovibrio strains, proving that this is a common receptor for different BALOs.

In conclusion, this study demonstrates that OmpF is widely recognized and used as a receptor for Bdellovibrio to recognize and attach specifically to E. coli prey cells. This was not the case for all the other bacterial species tested, however, indicating that other surface components act as receptors in those prey and suggesting that individual BALO strains possess a never-before-realized diversity in their prey recognition machinery, one that allows them to attach to and recognize diverse prey strains. While these results fit the scope of published data showing that BALOs have quite different and preferential activities against given prey (26–28), even when obtained from the same locale, future work in identifying these additional prey surface components and their roles in predation rates should be pursued.

### Ethical statement.

This article does not contain data from any studies with human participants or animals performed by any of the authors.

## References

[1] Dwidar M, Monnappa AK, Mitchell RJ. 2012. The dual probiotic and antibiotic nature of *Bdellovibrio bacteriovorus*. BMB Rep 45:71–78. doi:10.5483/BMBRep.2012.45.2.71. 22360883

[2] Bratanis E, Andersson T, Lood R, Bukowska-Faniband E. 2020. Biotechnological potential of *Bdellovibrio* and like organisms and their secreted enzymes. Front Microbiol 11:662. doi:10.3389/fmicb.2020.00662. 32351487 PMC7174725

[3] Dwidar M, Im H, Seo JK, Mitchell RJ. 2017. Attack-phase *Bdellovibrio bacteriovorus* responses to extracellular nutrients are analogous to those seen during late intraperiplasmic growth. Microb Ecol 74:937–946. doi:10.1007/s00248-017-1003-1. 28601973

[4] Im H, Dwidar M, Mitchell RJ. 2018. *Bdellovibrio bacteriovorus* HD100, a predator of Gram-negative bacteria, benefits energetically from *Staphylococcus aureus* biofilms without predation. ISME J 12:2090–2095. doi:10.1038/s41396-018-0154-5. 29849167 PMC6052163

[5] Jang H, Choi SY, Mun W, Jeong SH, Mitchell RJ. 2022. Predation of colistin- and carbapenem-resistant bacterial pathogenic populations and their antibiotic resistance genes in simulated microgravity. Microbiol Res 255:126941. doi:10.1016/j.micres.2021.126941. 34915266

[6] Matin A, Rittenberg SC. 1972. Kinetics of deoxyribonucleic acid destruction and synthesis during growth of *Bdellovibrio bacteriovorus* strain 109D on *Pseudomonas putida* and *Escherichia coli*. J Bacteriol 111:664–673. doi:10.1128/jb.111.3.664-673.1972. 4559819 PMC251338

[7] Stolp H, Starr MP. 1963. *Bdellovibrio bacteriovorus* gen. et sp. n., a predatory, ectoparasitic, and bacteriolytic microorganism. Antonie Van Leeuwenhoek 29:217–248. doi:10.1007/BF02046064. 14068454

[8] Li P, Lin H, Mi Z, Xing S, Tong Y, Wang J. 2019. Screening of polyvalent phage-resistant *Escherichia coli* strains based on phage receptor analysis. Front Microbiol 10:850. doi:10.3389/fmicb.2019.00850. 31105661 PMC6499177

[9] Zhao X, Cui Y, Yan Y, Du Z, Tan Y, Yang H, Bi Y, Zhang P, Zhou L, Zhou D, Han Y, Song Y, Wang X, Yang R. 2013. Outer membrane proteins ail and OmpF of *Yersinia pestis* are involved in the adsorption of T7-related bacteriophage Yep-phi. J Virol 87:12260–12269. doi:10.1128/JVI.01948-13. 24006436 PMC3807904

[10] Rabsch W, Ma L, Wiley G, Najar FZ, Kaserer W, Schuerch DW, Klebba JE, Roe BA, Laverde Gomez JA, Schallmey M, Newton SM, Klebba PE. 2007. FepA- and TonB-dependent bacteriophage H8: receptor binding and genomic sequence. J Bacteriol 189:5658–5674. doi:10.1128/JB.00437-07. 17526714 PMC1951831

[11] Silverman JA, Benson SA. 1987. Bacteriophage K20 requires both the OmpF porin and lipopolysaccharide for receptor function. J Bacteriol 169:4830–4833. doi:10.1128/jb.169.10.4830-4833.1987. 2820945 PMC213862

[12] Killmann H, Videnov G, Jung G, Schwarz H, Braun V. 1995. Identification of receptor binding sites by competitive peptide mapping: phages T1, T5, and phi 80 and colicin M bind to the gating loop of FhuA. J Bacteriol 177:694–698. doi:10.1128/jb.177.3.694-698.1995. 7836303 PMC176645

[13] Black PN. 1988. The fadL gene product of *Escherichia coli* is an outer membrane protein required for uptake of long-chain fatty acids and involved in sensitivity to bacteriophage T2. J Bacteriol 170:2850–2854. doi:10.1128/jb.170.6.2850-2854.1988. 3286621 PMC211212

[14] Im H, Kim D, Ghim C-M, Mitchell RJ. 2014. Shedding light on microbial predator–prey population dynamics using a quantitative bioluminescence assay. Microb Ecol 67:167–176. doi:10.1007/s00248-013-0323-z. 24272279

[15] Cho G, Kwon J, Soh SM, Jang H, Mitchell RJ. 2019. Sensitivity of predatory bacteria to different surfactants and their application to check bacterial predation. Appl Microbiol Biotechnol 103:8169–8178. doi:10.1007/s00253-019-10069-w. 31407038

[16] Maffei E, Shaidullina A, Burkolter M, Heyer Y, Estermann F, Druelle V, Sauer P, Willi L, Michaelis S, Hilbi H, Thaler DS, Harms A. 2021. Systematic exploration of Escherichia coli phage–host interactions with the BASEL phage collection. PLoS Biol 19:e3001424. doi:10.1371/journal.pbio.3001424. 34784345 PMC8594841

[17] Mizuno T, Mizushima S. 1987. Isolation and characterization of deletion mutants of ompR and envZ, regulatory genes for expression of the outer membrane proteins OmpC and OmpF in *Escherichia coli*. J Biochem 101:387–396. doi:10.1093/oxfordjournals.jbchem.a121923. 3294816

[18] Norioka S, Ramakrishnan G, Ikenaka K, Inouye M. 1986. Interaction of a transcriptional activator, OmpR, with reciprocally osmoregulated genes, *ompF* and *ompC*, of *Escherichia coli*. J Biol Chem 261:17113–17119. doi:10.1016/S0021-9258(19)76006-2. 3023382

[19] Ramakrishnan G, Ikenaka K, Inouye M. 1985. Uncoupling of osmoregulation of the *Escherichia coli* K-12 ompF gene from *ompB*-dependent transcription. J Bacteriol 163:82–87. doi:10.1128/jb.163.1.82-87.1985. 2989255 PMC219083

[20] Forst S, Delgado J, Ramakrishnan G, Inouye M. 1988. Regulation of *ompC* and *ompF* expression in *Escherichia coli* in the absence of *envZ*. J Bacteriol 170:5080–5085. doi:10.1128/jb.170.11.5080-5085.1988. 2846509 PMC211574

[21] Sato M, Machida K, Arikado E, Saito H, Kakegawa T, Kobayashi H. 2000. Expression of outer membrane proteins in *Escherichia coli* growing at acid pH. Appl Environ Microbiol 66:943–947. doi:10.1128/AEM.66.3.943-947.2000. 10698756 PMC91927

[22] Matsubara M, Mizuno T. 1999. EnvZ-independent phosphotransfer signaling pathway of the OmpR-mediated osmoregulatory expression of OmpC and OmpF in Escherichia coli. Biosci Biotechnol Biochem 63:408–414. doi:10.1271/bbb.63.408. 10192921

[23] Chakraborty S, Winardhi RS, Morgan LK, Yan J, Kenney LJ. 2017. Non-canonical activation of OmpR drives acid and osmotic stress responses in single bacterial cells. Nat Commun 8:1587. doi:10.1038/s41467-017-02030-0. 29138484 PMC5686162

[24] Shin H, Lee JH, Kim H, Choi Y, Heu S, Ryu S. 2012. Receptor diversity and host interaction of bacteriophages infecting *Salmonella enterica* serovar Typhimurium. PLoS One 7:e43392. doi:10.1371/journal.pone.0043392. 22927964 PMC3424200

[25] Schelling M, Conti S. 1986. Host receptor sites involved in the attachment of *Bdellovibrio bacteriovorus* and *Bdellovibrio stolpii*. FEMS Microbiol Lett 36:319–323. doi:10.1111/j.1574-6968.1986.tb01718.x.

[26] Van Essche M, Quirynen M, Sliepen I, Loozen G, Boon N, Van Eldere J, Teughels W. 2011. Killing of anaerobic pathogens by predatory bacteria. Mol Oral Microbiol 26:52–61. doi:10.1111/j.2041-1014.2010.00595.x. 21214872

[27] Jurkevitch E, Minz D, Ramati B, Barel G. 2000. Prey range characterization, ribotyping, and diversity of soil and rhizosphere *Bdellovibrio* spp. isolated on phytopathogenic bacteria. Appl Environ Microbiol 66:2365–2371. doi:10.1128/AEM.66.6.2365-2371.2000. 10831412 PMC110534

[28] Saralegui C, Herencias C, Halperin AV, de Dios-Caballero J, Pérez-Viso B, Salgado S, Lanza VF, Cantón R, Baquero F, Prieto MA, del Campo R. 2022. Strain-specific predation of *Bdellovibrio bacteriovorus* on *Pseudomonas aeruginosa* with a higher range for cystic fibrosis than for bacteremia isolates. Sci Rep 12:10523. doi:10.1038/s41598-022-14378-5. 35732651 PMC9217795

