# Supplementary material for: Outer Membrane Porin F in E. coli Is Critical for Effective Predation by Bdellovibrio
Source: Microbiol Spectr. 2022 Nov 29;10(6):e03094-22. doi: 10.1128/spectrum.03094-22 (PMC9769668; doi:10.1128/spectrum.03094-22)
Supplement: Supplemental file 1 — Supplemental methods, Tables S1 to S3, and Fig. S1 to S9. Download spectrum.03094-22-s0001.pdf, PDF file, 1.0 MB [file spectrum.03094-22-s0001.pdf]

Supplemental Information

for

Outer Membrane Porin F (OmpF) in *E. coli* is Critical for Effective Predation by *Bdellovibrio*

Running Title: Prey-Specific Recognition by Predatory Bacteria

Wonsik Mun,[1,†,*] Sumudu Upatissa,[1,†] Sungbin Lim, [1] Mohammed Dwidar [2,3,4,*] and Robert J. Mitchell [1,*]

[1] School of Biological Sciences, Ulsan National Institute of Science and Technology (UNIST), Ulsan 44919, South Korea

[2] Cleveland Clinic Lerner College of Medicine, Case Western Reserve University, Cleveland, OH 44195, USA

[3] Department of Cardiovascular & Metabolic Sciences, Lerner Research Institute, Cleveland Clinic, Cleveland, OH 44195, USA

[4] Center for Microbiome and Human Health, Lerner Research Institute, Cleveland Clinic, Cleveland, OH 44195, USA

[†] - These authors contributed equally

Correspondence

W. M. – wmun@unist.ac.kr

M. D. – dwidarm@ccf.org

R. J. M. – esgott@unist.ac.kr

Supplementary methods

Bacterial strains and culturing conditions

All the bacterial strains used in this study are listed in Table S1. Each of the prey and their isogenic mutants were routinely propagated on lysogen broth (LB) agar plates. Fresh single colonies were cultured in LB broth, incubated overnight in a shaking incubator at 37 ºC, centrifuged (5000 x g, 15 min) and the pellet was re-suspended in the predation media. All the predatory strains were routinely grown as described previously (1, 2) using *E. coli* MG1655/pUCDK as the prey.

Bioluminescence assay to monitor the predation kinetics

The *E. coli* prey strains were rendered bioluminescent by transforming them with pGEN-luxCDABE (3), a gift from Harry Mobley (Addgene plasmid # 44918; http://n2t.net/addgene:44918; RRID: Addgene_44918). Overnight cultures of these prey strains were grown in LB broth supplemented with 100 µg/ml ampicillin at 37 ºC before being diluted to an optical density (OD_{600nm}) of 0.05 in dilute nutrient broth (DNB; 1/10 NB) containing 3 mM $MgCl_2$ and 2 mM $CaCl_2$. The predator was grown as described above, filtered, and diluted two-fold in 25 mM HEPES (with 3 mM $MgCl_2$ and 2 mM $CaCl_2$, pH 7.2). The predator and prey cell densities in each sample were determined using top agar plates and colony counts, respectively, as described previously (4), and were used to calculate the predator-prey ratio (PPR). The predator and prey preparations were mixed 1:1 (v:v; 100 µl each) in the wells of a 96-well plate (white, Greiner, USA) and the bioluminescence was measured every ten minutes as described previously (5).

Prey viability assessment

For these experiments, the prey was diluted to OD 0.05 in DNB. *B. bacteriovorus* 109J was grown as above and diluted in HEPES buffer (with 3 mM MgCl2 and 2 mM CaCl2, pH 7.2). The predator dilutions and prey suspensions were mixed (1:1 (v:v)) so that the predator-to-prey ratio was 6.25, 25 or 100. Each sample was then incubated with shaking incubator (250 rpm) at 30 ºC for 1 hour, after which the viability of the prey was determined using plate counts on LB agar plates.

Complementation of *E. coli* BW25113 Δ*ompF* and *E. coli* BW25113 Δ*ompC*

The pCA24N plasmid was linearized for In-Fusion® cloning using primers pCA ir and pCA if primers (Table S2). The *ompF* gene was amplified from *E. coli* MG1655 with its flanking

regions using primers pCA-Omp F and pCA-Omp R primers (Table S2). After purifying both the vector and insert, they were recombined using the In-Fusion® manufacturer's suggested protocol, generating the complementation plasmid, pCA-*ompF*. This plasmid was transformed into *E. coli* DH5α cells, which were then grown on LB agar plates containing chloramphenicol (35 µg/ml). The plasmid from an individual colony was purified and sequenced using the pCA24 seq F and pCA24 seq R primer set (Table S2). Once the sequence was verified, the plasmid (pCA-*ompF*) was transformed into the *E. coli* BW25113 strains.

The same was used to also construct the pCA-*ompC* plasmid. For this, the *ompC* gene was amplified from wild-type *E. coli* BW25113 with additional flanking region using primers pCA-*ompC* F and pCA-*ompC* R (Table S2). After construction, transformation and purification of the plasmid as above, its sequence was confirmed using primers pCA24 seq F and pCA24 seq R (Table S2).

Microscopic analyses of predation

Using *E. coli* S17 λ-pir as the donor, plasmid pMQ414, which expresses the tdTomato fluorescence protein (6), was transferred into *B. bacteriovorus* 109J through conjugation. The predation tests were conducted using this fluorescent predatory strain and a synchronized attack, to ensure many of the prey were attacked at the same time, as described previously (7). Briefly. after growth of the fluorescent predator using the same protocol as described above, it was concentrated 10-fold by centrifugation (7000 x g, 15 min) and resuspended in fresh HEPES buffer (with 3 mM $MgCl_2$ and 2 mM $CaCl_2$, pH 7.2). For these predation assays, wild-type *E. coli* BW25113 and three isogenic mutants (*i.e.*, Δ*ompF*, Δ*ompR* and Δ*envZ*) were all used as prey. Each was grown overnight as above, centrifuged (7000 x g, 15 min) and resuspended in HEPES buffer to an OD_{600nm} of 4.0. These samples (*i.e.*, predator and prey solutions) were stored at 30 ºC for 10 minutes before they were mixed 1:1 (v:v). The mixed cultures were incubated in a shaking incubator at 30 ºC and samples were taken at set times (*i.e.*, 0, 20 and 60 min) and fixed with an equal volume of 8% (w/v) paraformaldehyde (PFA) prepared in the same HEPES buffer. The fixed samples were stored at 4 ºC until being observed by confocal microscopy. The number of each prey cell type (*i.e.*, free prey, prey with a predator attached or bdelloplast) were counted and analyzed at the indicated time points.

Growth of *E. coli* at Higher Osmolalities

To study the impact of the medium osmolality on predation rates, *E. coli* BW25113 and *E. coli* JW0912 (Δ*ompF*) were grown in LB medium prepared without NaCl addition. Before autoclaving, NaCl was added to a final concentration of 0, 0.25, 0.5 and 1% (w:v), generating osmolalities of 78, 162, 256 and 427 mOsm/kg, respectively, for each medium. Growth of the prey was conducted as described above. After growth overnight, the prey cells were pelleted (5000 x g, 15 min), washed in sterile HEPES to remove the salts (1, 8) and resuspended in fresh DNB to an OD of 0.05, as described previously (9).

P1 transduction to generate *ompF* knockouts in the different *E. coli* strains

E. coli JW0912 (*i.e.*, the isogenic *E. coli* BW25113 Δ*ompF* strain from the KEIO collection (10)) was used as the donor strain for the preparation of the P1 phage lysate. The host *E. coli* strains used were *E. coli* BL21(DE3), *E. coli* MG1655 and *E. coli* DSM 613. After transduction according to the previously published protocol (11), successful mutants were positively selected using kanamycin (35 µg/ml) plates containing 5 mM sodium citrate. Deletion of the *ompF* gene in each *E. coli* strain was confirmed by PCR using the primers listed in Table S2.

Constructing *ompF* knockout mutants in the non-*E. coli* prey

Deletion of the *ompF* gene in *E. fergusonii* and *ompK35* (*ompF* homologue (68% identity based on amino acid sequence)) in *K. pneumoniae* was achieved using a suicide plasmid as described previously (12). Briefly, a suicide plasmid harboring a *sacB* gene cassette, kanamycin resistance gene cassette, the R6K replication origin and a RP4-oriT was constructed. Sets of primers (Table S2) were used to amplify approximate 1 kb homologous recombination arms flanking the genes in *K. pneumoniae* and *E. fergusonii*. These homologous recombination arms included the first and the last 20~30 amino acids of the gene in each case. Each was then fused through a third PCR reaction and ligated to the suicide plasmid using the In-Fusion® HD Cloning kit (Clontech). The ligated plasmid was transformed into *E. coli* S17 λ-pir through chemical transformation and transferred via conjugation to the corresponding recipient strain (*E. fergusonii* or *K. pneumoniae*). *E. fergusonii* ATCC 35473 and both *K. pneumoniae* WGLW1 and WGLW2 are naturally resistant to ampicillin, allowing us to screen the conjugants on LB agar plates containing 100 µg/mL ampicillin (to select against the *E. coli* S17 λ-pir donor cells) and 50 µg/mL kanamycin (to select for merodiploids). One merodiploid mutant in each case was selected

and grown on an LSW-sucrose agar plate (tryptone 10 g/L, yeast extract 5 g/L, glycerol 5 ml/L, NaCl 0.4 g/l, sucrose 100 g/L and agar 20 g/L) (12) to generate a double crossover mutant. One colony was then selected and grown on LB agar with no antibiotics. Loss of the conjugated plasmid in this strain was confirmed by PCR using the primers listed in Table S2, as well as phenotypically as the mutant was unable to grow in presence of kanamycin.

To generate an *ompF* deletion in *S. enterica* LT2, lambda red recombineering was employed as previously described (10, 13). The resulting *ompF* knockout clone had its gene replaced with the kanamycin resistance cassette, leaving only the first 30 and last 18 amino acids of the host *ompF* gene.

Isolation of novel BALO strains

B. bacteriovorus strains EY2.3, EY3.2, DH1 and SM1 (Table S1) are all environmental isolates. *B. bacteriovorus* EY2.3 and EY3.2 were isolated from the Eonyang Wastewater Treatment Plant (Eonyang, South Korea), while *B. bacteriovorus* DH1 and SM1 were isolated from forest soil in Ulju-gun, Ulsan, South Korea. To isolate each, samples from these sites were gently mixed with 20 ml of HEPES (3 mM MgCl2 and 2 mM CaCl2, pH 7.2) in a benchtop mixer for an hour. After centrifuging the samples (1000 x g for 5 minutes) to settle down large particulates, the supernatants were collected, and 1 ml was mixed with 10 ml of prepared molten top agar (DNB supplemented with the salts and prey (*E. coli* MG1655)). The plates were incubated at 30 °C until clear plaques were visualized. Individual plaques were then collected and sub-cultured with freshly prepared prey to isolate the predators. Each predatory strain was identified as being *Bdellovibrio* based on their 16S rDNA sequence, which had high homology to that of *B. bacteriovorus* 109J (Table S3).

Reproducibility and statistical analysis

Unless specified, each experiment was performed in triplicate and the standard deviations are presented on the graphs as error bars. Normal distribution of each dataset was verified using the Shapiro-Wilk test. None of the samples showed a substantial departure from the normality ($p > 0.05$) and, thus, the student t-test was used to evaluate statistical significance between two sets of data. Significance is indicated within the graphs using: a - $p < 0.05$; b - $p < 0.01$; c - $p < 0.001$.

Supplementary figures and tables

Table S1. Strains and plasmids used in this study

Prey Bacterial Strains	Description	Gene Function	Ref
E. coli BW25113	Wild-type strain	-	(10)
E. coli JW5195	Isogenic Δ*tonB*	Component of the energy transducing Ton system	(10)
E. coli JW5086	Isogenic Δ*fepA*	Ferric enterobactin outer membrane transporter	(10)
E. coli JW2341	Isogenic Δ*fadL*	Long-chain fatty acid outer membrane channel	(10)
E. coli JW0146	Isogenic Δ*fhuA*	Ferrichrome outer membrane transporter	(10)
E. coli JW0940	Isogenic Δ*ompA*	Outer membrane protein A	(10)
E. coli JW2203	Isogenic Δ*ompC*	Outer membrane porin C	(10)
E. coli JW0912	Isogenic Δ*ompF*	Outer membrane porin F	(10)
E. coli JW1312	Isogenic Δ*ompG*	Outer membrane porin G	(10)
E. coli JW3846	Isogenic Δ*ompL*	Putative outer membrane porin L	(10)
E. coli JW1371	Isogenic Δ*ompN*	Outer membrane porin N	(10)
E. coli JW3368	Isogenic Δ*ompR*	EnvZ/OmpR two-component response regulator	(10)
E. coli JW0554	Isogenic Δ*ompT*	Omptin family outer membrane protease	(10)
E. coli JW1248	Isogenic Δ*ompW*	Outer membrane protein W	(10)
E. coli JW0799	Isogenic Δ*ompX*	Outer membrane protein X	(10)
E. coli JW3367	Isogenic Δ*envZ*	EnvZ/OmpR two-component sensor histidine kinase	(10)
E. coli MG1655	Wild-type strain	-	
E. coli MG1655 Δ*ompF*	Isogenic Δ*ompF*	Outer membrane porin F	This study
E. coli BL21(DE3)	Wild-type strain	-	
E. coli BL21(DE3) Δ*ompF*	Isogenic Δ*ompF*	Outer membrane porin F	This study
E. coli DSM 613	Wild-type strain	-	
E. coli DSM 613 Δ*ompF*	Isogenic Δ*ompF*	Outer membrane porin F	This study
E. fergusonii ATCC 35469	Wild-type strain	-	(14)
E. fergusonii ATCC 35469 Δ*ompF*	Isogenic Δ*ompF*	OmpF homologue	This study
Salmonella enterica LT2	Wild-type strain		(15)
S. enterica LT2 Δ*ompF*	Isogenic Δ*ompF*	OmpF homologue	This study
Klebsiella pneumoniae WGLW1	Wild-type strain	-	
K. pneumoniae WGLW1 Δ*ompK35*	Isogenic ⊿ompK35	OmpF homologue	This study
Klebsiella pneumoniae WGLW2	Wild-type strain	-	
K. pneumoniae WGLW2 Δ*ompK35*	Isogenic ⊿ompK35	OmpF homologue	This study
**Predatory Bacterial Strains**		**Characteristics**	
Bdellovibrio bacteriovorus 109J	Wild-type strain	ATCC 43826	
Bdellovibrio bacteriovorus HD100	Wild-type strain	DSM 50701	
Bdellovibrio str. EY2.3	Wild-type strain	New isolate from the Eonyang wastewater treatment plant	This study
Bdellovibrio str. EY3.3	Wild-type strain	New isolate from the Eonyang wastewater treatment plant	This study
Bdellovibrio str. DH1	Wild-type strain	New isolate from forest soil near Eonyang, South Korea	This study
Bdellovibrio str. SM1	Wild-type strain	New isolate from forest soil near Eonyang, South Korea	This study
**Plasmids**			
pGEN-luxCDABE		Expresses *luxCDABE*; Generates bioluiminescence	(3)
pCA24N		Empty vector	(16)
pCA-*ompF*		Expresses *ompF*: complementation	This study
pCA-*ompC*		Expresses *ompC*: complementation	This study

Table S2. List of primers used in this study

Primer	Sequence	Purpose
pCA ir	ACGCAGGAAAGAACATGTGAG	Constructing *ompF* complementation plasmid
pCA if	GACCTGCAGCCAAGCTTA	Constructing *ompF* complementation plasmid
pCA-Omp F	TCACATGTTCTTTCCTGCGTTGCTGTAAATA TCATCACGTCTC	Constructing *ompF* complementation plasmid
pCA-Omp R	ATTAAGCTTGGCTGCAGGTCTTAGAACTGG TAAACGATACCCA	Constructing *ompF* complementation plasmid
pCA-OmpC F	TCACATGTTCTTTCCTGCGTATTCAGTGCTG TCAAATACTTAAGA	Constructing *ompC* complementation plasmid
pCA-OmpC R	ATTAAGCTTGGCTGCAGGTCTTAGAACTGG TAAACCAGACC	Constructing *ompC* complementation plasmid
pCA24 seq F	TGGAAAAACGCCAGCAAC	Sequence verification of *ompF* and *ompC* complementation plasmids
pCA24 seq R	CTGAACAAATCCAGATGGAGTTC	Sequence verification of *ompF* and *ompC* complementation plasmids
Kn R	TCAGAAGAACTCGTCAAGAAG	Amplifying the suicide plasmid used for knocking out *ompF* in *E. fergusonii* and *K. pneumoniae*
SacB F	GGAAAATAGACCAGTTGCAATCC	Amplifying the suicide plasmid used for knocking out *ompF* in *E. fergusonii* and *K. pneumoniae*
Ef-ompF up F	GTGGTTCCCAGCGTGAAG	Cloning upstream homology arm for *E. fergusonii ompF*
Ef-ompF up R	TGCGGTACCTGCTACCAA	Cloning upstream homology arm for *E. fergusonii ompF*
Ef-ompF Dn F	TCGTCCCTGCTCTGTTGGTAGCAGGTACCG CAGGCGTAGGTTCTGACGAT	Cloning downstream homology arm for *E. fergusonii ompF*
Ef-ompF Dn R	CGAGCAAACCCCTGGTAA	Cloning downstream homology arm for *E. fergusonii ompF*
Kn-Ef-ompF F	TTCTTGACGAGTTCTTCTGAGTGGTTCCCA GCGTGAAG	Fusing the two homology arms for knocking out *ompF* in *E. fergusonii*
Sac-Ef-ompF R	TTGCAACTGGTCTATTTTCCCGAGCAAACC CCTGGTAA	Fusing the two homology arms for knocking out *ompF* in *E. fergusonii*
Ef-ompF seq up F	CAGAATTTATTGGCGGCAGT	Sequence verification of *E. fergusonii* Δ*ompF*
Ef-ompF confirm F	ACGAAGACGGAAAAACCGT	Sequence verification of *E. fergusonii* Δ*ompF*
Ef-ompF confirm R	GCTGGCAATGATCAGCTCT	Sequence verification of *E. fergusonii* Δ*ompF*
Kp-ompF up F	GGATCTCTCCTCTGAGCAC	Cloning upstream homology arm for constructing *K. pneumoniae* Δ*ompF*
Kp-ompF up R	GATTTCTGCAGCGTTGGCT	Cloning upstream homology arm for constructing *K. pneumoniae* Δ*ompF*
Kp-ompF Dn F	TGGTAGCCGGTGCAGCCAACGCTGCAGAA ATCTTCAACCAGCTGGACGACA	Cloning downstream homology arm for constructing *K. pneumoniae* Δ*ompF*
Kp-ompF Dn R	CAAAAGCGCGTAGCCCTTC	Cloning downstream homology arm for constructing *K. pneumoniae* Δ*ompF*
Kn-Kp-ompF F	TTCTTGACGAGTTCTTCTGAGGATCTCTCCT CTGAGCAC	Fusing the two homology arms for knocking out *ompF* in *K. pneumoniae*
Sac-Kp-ompF R	TTGCAACTGGTCTATTTTCCCAAAAGCGCG TAGCCCTTC	Fusing the two homology arms for knocking out *ompF* in *K. pneumoniae*
Kp-ompF seq up F	TAGATAAGTAATGGCGTTTGCC	Sequence verification of *K. pneumoniae* Δ*ompF*
Kp-ompF confirm F	TTATCACCGCTGATTTTGCC	Sequence verification of *K. pneumoniae* Δ*ompF*
Kp-ompF confirm R	GTCTACAGTCTCCTGATCCG	Sequence verification of *K. pneumoniae* Δ*ompF*
Sa-ompF-Kn F	GCAAAATCCTGGCAGCGGTGATCCCTGCCC TGCTGGCTGCTGCAACCGCAATTCCGGGGA TCCGTCGACC	Knocking out *ompF* in *S. enterica* through lambda red recombineering
Sa-ompF-Kn R	TCAGAACTGGTAAGTAATACCGACAGCCG CCTGATCGTCGGTGCCAACGTTGTAGGCTG GAGCTGCTTCG	Knocking out *ompF* in *S. enterica* through lambda red recombineering

Table S3. Identification of the new predatory isolates

Predatory Strain	Isolation Locale	Homology	Gene Region[a]
Bdellovibrio str. EY2.3	Eonyang WWTP	98.92% (1378/1393 bp)	1036992 ~ 1038386
Bdellovibrio str. EY3.3	Eonyang WWTP	98.92% (1378/1393 bp)	1036992 ~ 1038386
Bdellovibrio str. DH1	Forest Soil	99.73% (1453/1457 bp)	1036920 ~ 1038376
Bdellovibrio str. SM1	Forest Soil	100% (1457/1457 bp)	1036920 ~ 1038376

[a] – Based on the published 16S rDNA gene sequence for *B. bacteriovorus* 109J (NCBI Accession No. NZ_CP007656.1)

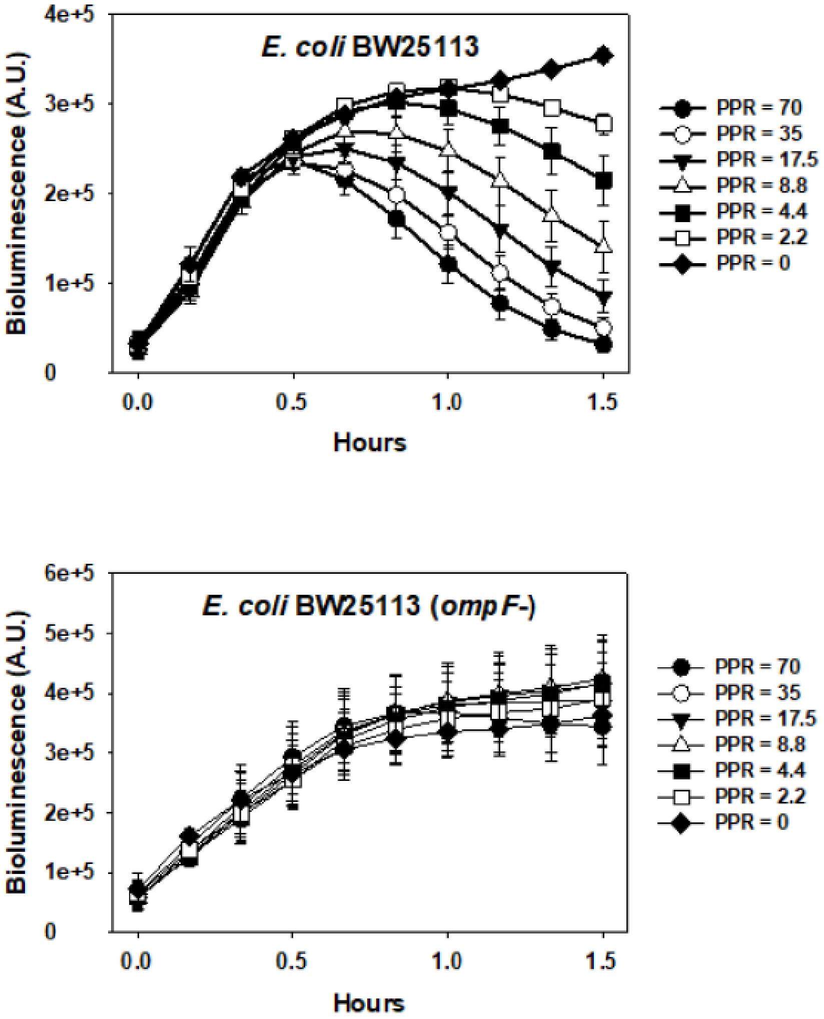

Figure S1. Plots showing the predation kinetics over 1.5 hours with *E. coli* BW25113 (Top) and its isogenic Δ*ompF* mutant strain, *E. coli* JW0912 (Bottom). Both strains harbored pGen-LuxCDABE (Table S1), making them bioluminescent. The results show the clear dose-dependent loss in bioluminescence according to the initial PPR value for *E. coli* BW25113, while the isogenic ΔompF mutant showed almost no loss, although a PPR of 70 showed a downward trend, indicating this mutant strain is not truly resistant (Figure S2). (n = 3)

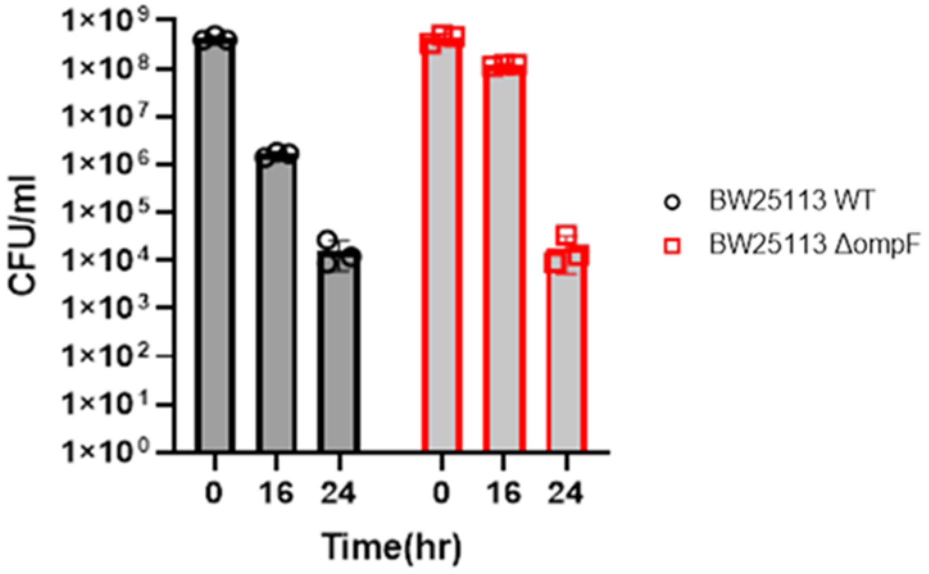

Figure S2. Although the Δ*ompF* isogenic mutant of *E. coli* BW25113 is not predated efficiently by *B. bacteriovorus* 109J, it is not resistant. This graph shows that predation of the *E. coli* Δ*ompF* mutant was still not very apparent (< 1-log reduction) after 16 h but, by 24 h, the prey viability was no different than that of wild-type E. coli BW25113, both reduced by more than 4-log. (n = 3)

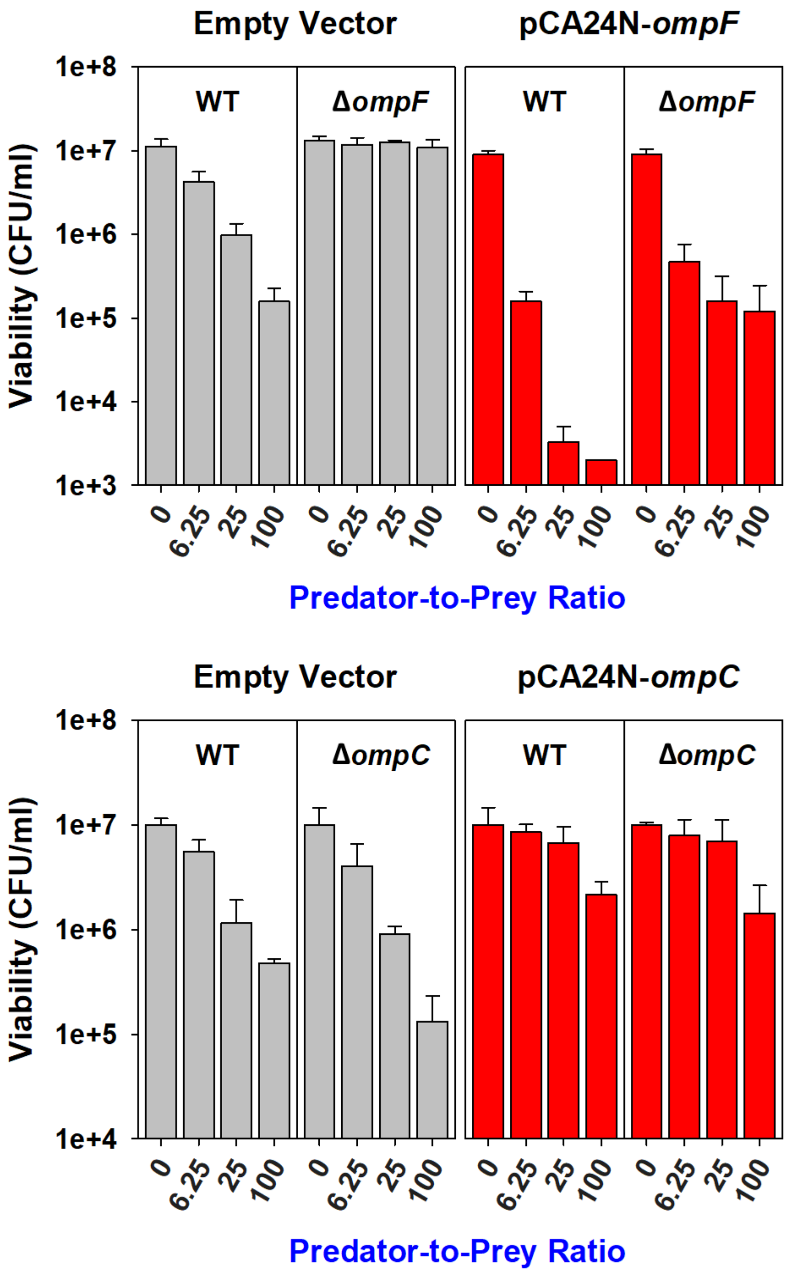

Figure S3. Prey viability results used in plotting Figure 1b, showing expression of a functional *ompF* gene increases predation rates. Complementation of the Δ*ompF* knock-out led to similar predation rates as the wild-type *E. coli* BW25113 while over-expression of the *ompF* gene in the wild-type *E. coli* BW25113 background led to significantly better predation rates. In contrast, as shown in the bottom graph, loss of the *ompC* gene increased predation, while over-expression or complementation of this gene in *E. coli* suppressed predatory activities against this prey. The viabilities were measured after one hour of predation. (n = 3)

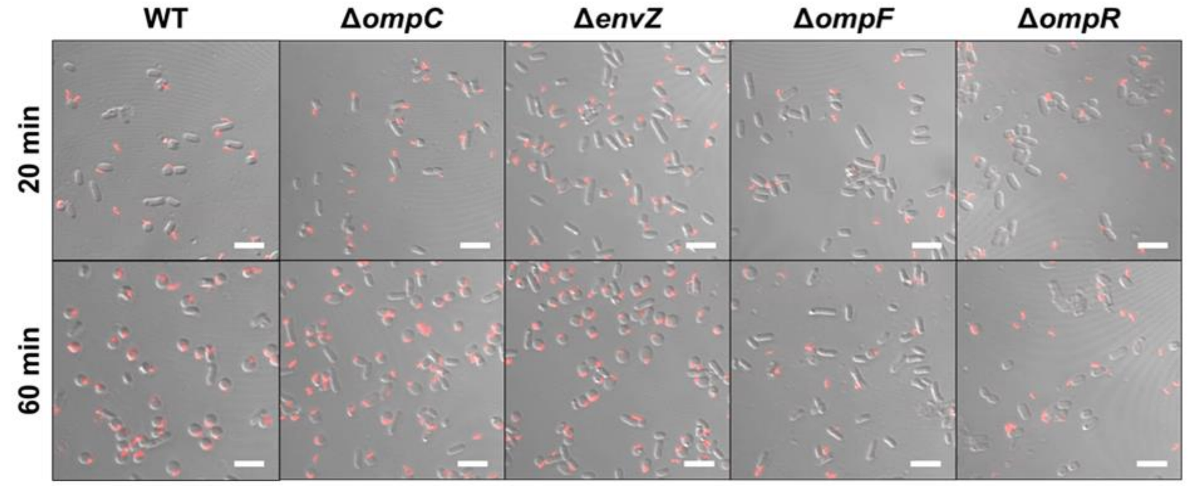

Figure S4. Representative microscopic images of the different isogenic mutants of *E. coli* BW25113 during predation. The predator (*B. bacteriovorus* 109J) expresses tdTomato, making it fluoresce red. Attachment was defined as a rod-shaped prey with the predator clearly attached to it, while bdelloplast formation was defined by prey cells that were spherical in shape with a fluorescent predator present within them. Scale bar – 5 µm

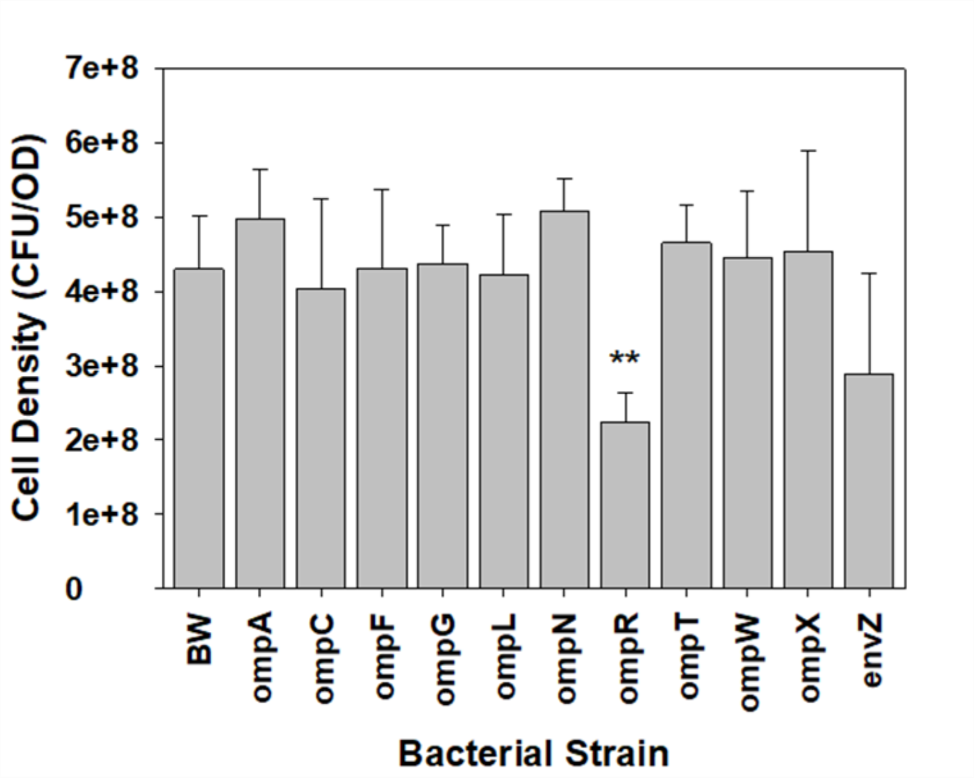

Figure S5. Cell densities of the different *E. coli* BW25113 isogenic mutants. The results show all but *E. coli* BW25113 Δ*ompR* have similar densities per OD. These values were used to calculate the PPR values. ** - p , 0.01 (n = 3)

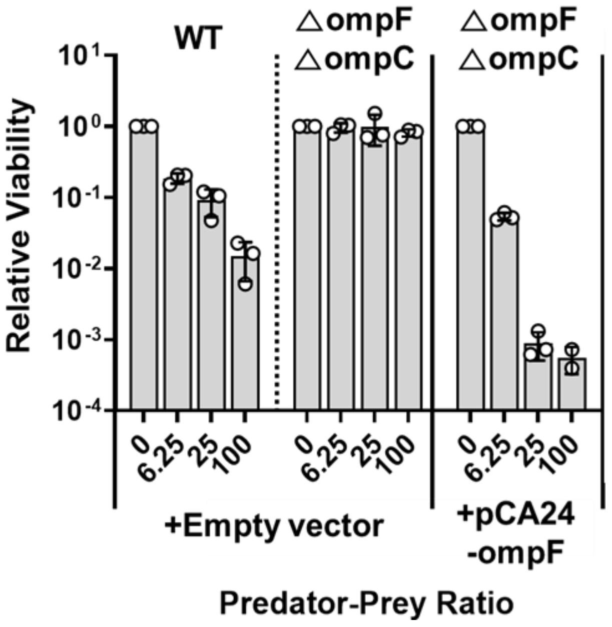

Figure S6. **Predation of a double (Δ*ompC* Δ*ompF*) mutant of *E. coli* BW25113**. Much like *E. coli* JW0912 (Δ*ompF*), this double mutant is predated slowly. Complementation with a functional *ompF* gene significantly improves predation against this prey, illustrating the importance of OmpF in recognition of *E. coli*. (n = 3)

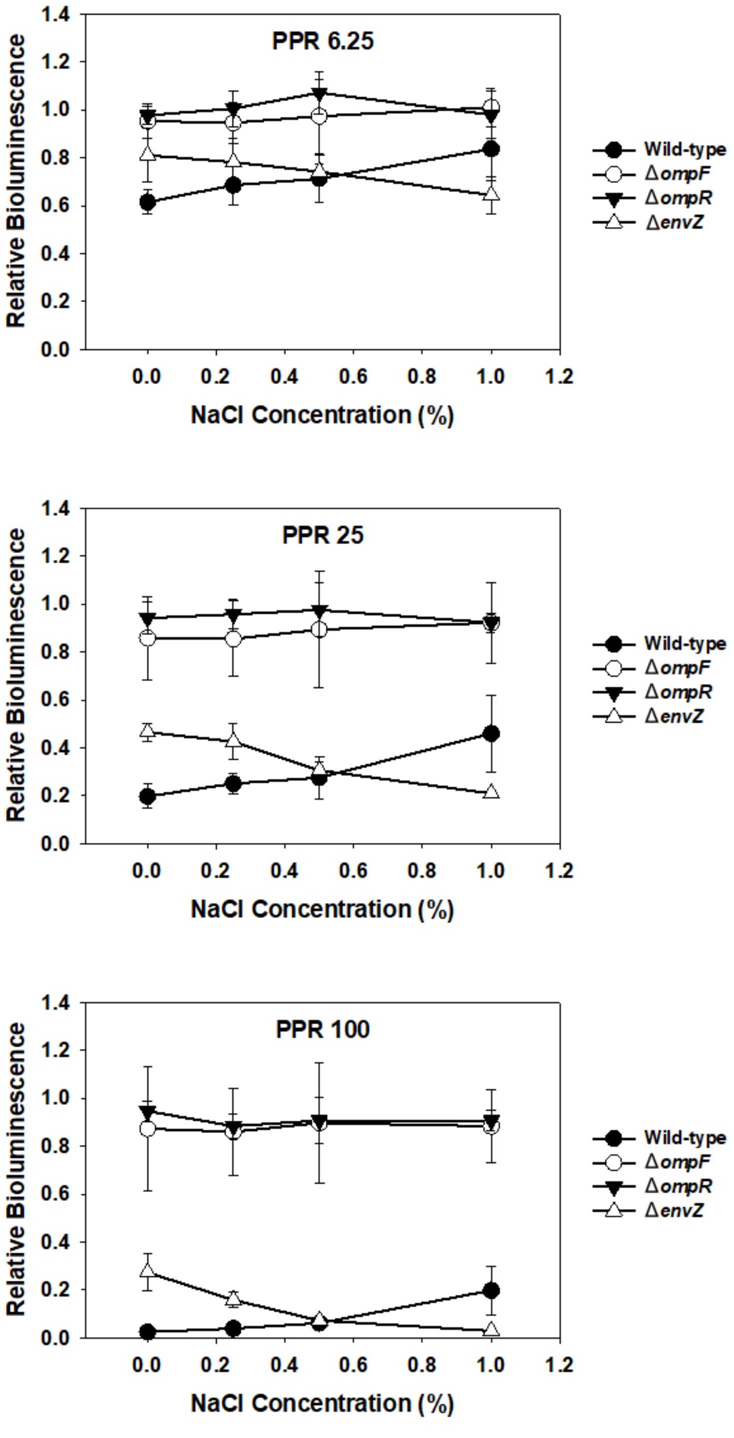

Figure S7. Impacts of NaCl during growth on the predation of the isogenic mutants of *E. coli* BW25113/pGen-luxCDABE. The data presented here is shown relative against the unpredated control (PPR = 0) at one hour. Much like *E. coli* JW0912 (Δ*ompF*), *E. coli* JW3368 (Δ*ompR*) is also predated slowly. In contrast, *E. coli* JW3367 (Δ*envZ*) is predated better when the NaCl concentration in the LB medium during growth was higher. (n = 3)

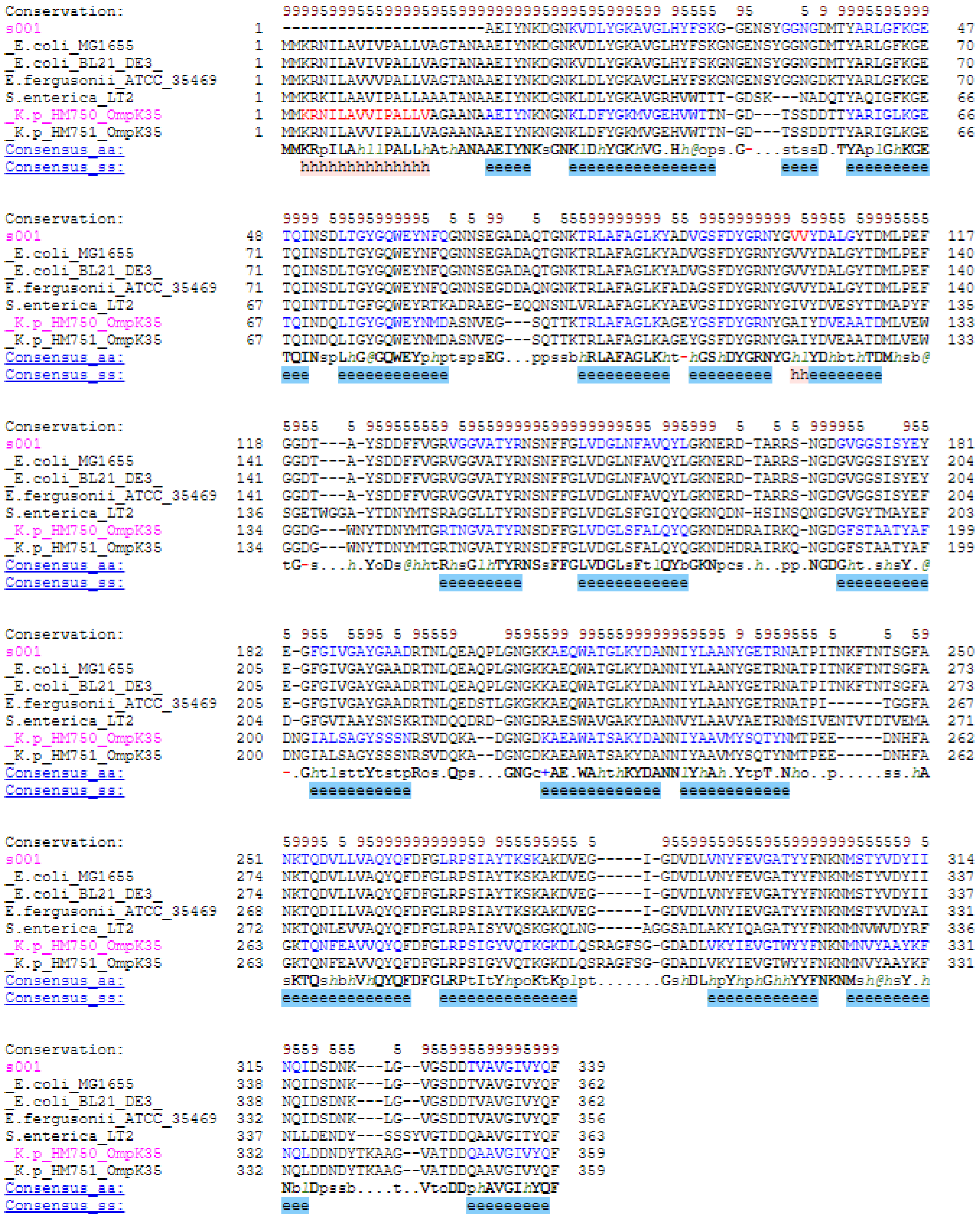

Figure S8. **Multiple sequence alignment of the different OmpF (and OmpK35) proteins**. The alignment was performed using the PROMALS3D server using the default parameters (17,18). The structure of OmpF of *E. coli* MH225 was retrieved from RCSB Protein Data Bank (https://www.rcsb.org) and used as the reference structure (Sequence “s001”). The first line in each block shows conservation indices for positions with a conservation index above 5. Each representative sequence has a magenta name and is colored according to PSIPRED (19)

(red: alpha-helix, blue: beta-strand). A representative sequence and the immediate sequences below it with black names form a closely related group. The last two lines show consensus amino acid sequence (Consensus_aa) and consensus predicted secondary structures (Consensus_ss). Consensus predicted secondary structure symbols: alpha-helix: h; beta-strand: e.

Conserved amino acids are in bold and uppercase letters; aliphatic (I, V, L): l; aromatic (Y, H, W, F): @; hydrophobic (W, F, Y, M, L, I, V, A, C, T, H): h; alcohol (S, T): o; polar residues (D, E, H, K, N, Q, R, S, T): p; tiny (A, G, C, S): t; small (A, G, C, S, V, N, D, T, P): s; bulky residues (E, F, I, K, L, M, Q, R, W, Y): b; positively charged (K, R, H): +; negatively charged (D, E): -; charged (D, E, K, R, H): c.

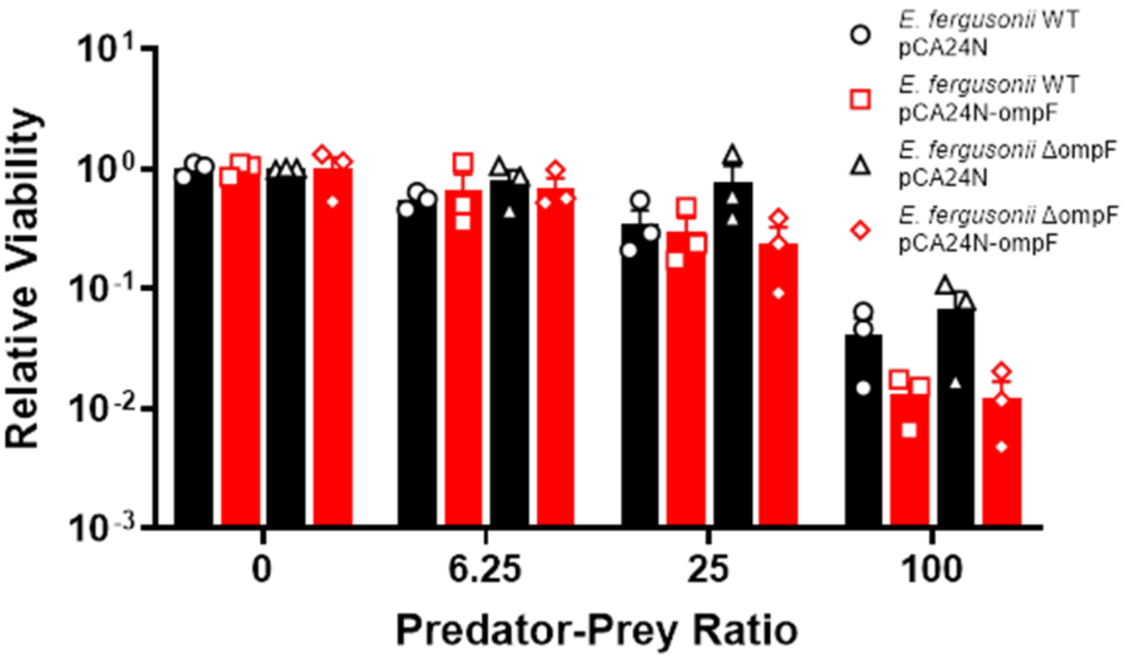

Figure S9. Expression of *E. coli* OmpF in *E. fergusonii* increases predation of this prey. As noted in Figure 2b, loss of the *ompF* homologue in *E. fergusonii* had no obvious impact on predation rates. However, expressing *ompF* from *E. coli* BW25113 within this prey led to better predation efficiencies, in both the wild-type and Δ*ompF* genetic backgrounds. These results show OmpF in *E. coli* is being recognized by *B. bacteriovorus* 109J. (n = 3)

References

1. Jang H, Mun W, Choi SY, Mitchell RJ, Khursigara CM. 2022. Use of Resazurin To Rapidly Enumerate *Bdellovibrio* and Like Organisms and Evaluate Their Activities. Microbiol Spectr 10.
2. Im H, Kwon H, Cho G, Kwon J, Choi SY, Mitchell RJ. 2019. Viscosity has dichotomous effects on *Bdellovibrio bacteriovorus* HD100 predation. Environ Microbiol 21:4675-4684.
3. Lane MC, Alteri CJ, Smith SN, Mobley HLT. 2007. Expression of flagella is coincident with uropathogenic *Escherichia coli* ascension to the upper urinary tract. Proc Natl Acad Sci 104:16669-16674.
4. Cho G, Kwon J, Soh SM, Jang H, Mitchell RJ. 2019. Sensitivity of predatory bacteria to different surfactants and their application to check bacterial predation. Appl Microbiol Biotechnol 103:8169-8178.
5. Im H, Kim D, Ghim C-M, Mitchell RJ. 2013. Shedding Light on Microbial Predator–Prey Population Dynamics Using a Quantitative Bioluminescence Assay. Microb Ecol 67:167-176.
6. Mukherjee S, Brothers KM, Shanks RMQ, Kadouri DE, Liu SJ. 2016. Visualizing *Bdellovibrio bacteriovorus* by Using the tdTomato Fluorescent Protein. Appl Environ Microbiol 82:1653-1661.
7. Dwidar M, Jang H, Sangwan N, Mun W, Im H, Yoon S, Choi S, Nam D, Mitchell RJ. 2020. Diffusible Signaling Factor, a Quorum-Sensing Molecule, Interferes with and Is Toxic Towards *Bdellovibrio bacteriovorus* 109J. Microb Ecol 81:347-356.
8. Im H, Son S, Mitchell RJ, Ghim CM. 2017. Serum albumin and osmolality inhibit *Bdellovibrio bacteriovorus* predation in human serum. Sci Rep 7:5896.
9. Im H, Kim D, Ghim CM, Mitchell RJ. 2014. Shedding light on microbial predator-prey population dynamics using a quantitative bioluminescence assay. Microb Ecol 67:167-76.
10. Baba T, Ara T, Hasegawa M, Takai Y, Okumura Y, Baba M, Datsenko KA, Tomita M, Wanner BL, Mori H. 2006. Construction of *Escherichia coli* K-12 in-frame, single-gene knockout mutants: the Keio collection. Mol Syst Biol 2:2006 0008.
11. Thomason LC, Costantino N, Court DL. 2014. *E. coli* Genome Manipulation by P1 Transduction. Curr Protocols Mol Biol 79.
12. Howery KE, Rather PN. 2019. Allelic Exchange Mutagenesis in *Proteus mirabilis*. Methods Mol Biol 2021:77-84.
13. Datsenko KA, Wanner BL. 2000. One-step inactivation of chromosomal genes in *Escherichia coli* K-12 using PCR products. Proc Natl Acad Sci U S A 97:6640-5.
14. Farmer JJ, Fanning GR, Davis BR, O'Hara CM, Riddle C, Hickman-Brenner FW, Asbury MA, Lowery VA, Brenner DJ. 1985. *Escherichia fergusonii* and *Enterobacter taylorae*, two new species of *Enterobacteriaceae* isolated from clinical specimens. J Clin Microbiol 21:77-81.
15. McClelland M, Sanderson KE, Spieth J, Clifton SW, Latreille P, Courtney L, Porwollik S, Ali J, Dante M, Du F, Hou S, Layman D, Leonard S, Nguyen C, Scott K, Holmes A, Grewal N, Mulvaney E, Ryan E, Sun H, Florea L, Miller W, Stoneking T, Nhan M, Waterston R, Wilson RK. 2001. Complete genome sequence of *Salmonella enterica* serovar Typhimurium LT2. Nature 413:852-856.
16. Kitagawa M, Ara T, Arifuzzaman M, Ioka-Nakamichi T, Inamoto E, Toyonaga H, Mori H. 2006. Complete set of ORF clones of *Escherichia coli* ASKA library (A Complete Set of *E. coli* K-12 ORF Archive): Unique Resources for Biological Research. DNA Res 12:291-299.
17. Pei J, Kim BH, Grishin NV. 2008. PROMALS3D: a tool for multiple protein sequence and structure alignments. Nucleic Acids Res 36:2295-300.
18. Pei J, Grishin NV. 2014. PROMALS3D: multiple protein sequence alignment enhanced with evolutionary and three-dimensional structural information. Methods Mol Biol 1079:263-71.
19. McGuffin LJ, Bryson K, Jones DT. 2000. The PSIPRED protein structure prediction server. Bioinformatics 16:404-5
